# Participatory Methods for Systems Modeling of Youth Mental Health: Implementation Protocol

**DOI:** 10.2196/32988

**Published:** 2022-02-07

**Authors:** Louise Freebairn, Jo-An Occhipinti, Yun Ju C Song, Adam Skinner, Kenny Lawson, Grace Yeeun Lee, Samuel J Hockey, Samantha Huntley, Ian B Hickie

**Affiliations:** 1 Brain and Mind Centre Faculty of Medicine and Health University of Sydney Camperdown Australia; 2 Computer Simulation & Advanced Research Technologies (CSART) Sydney Australia; 3 Research School of Population Health Australian National University Canberra Australia

**Keywords:** participatory system modeling, youth mental health, co-design, public health systems research, mental health services

## Abstract

**Background:**

Despite significant investment, mental health issues remain a leading cause of death among young people globally. Sophisticated decision analysis methods are needed to better understand the dynamic and multisector drivers of youth mental health. System modeling can help explore complex issues such as youth mental health and inform strategies to effectively respond to local needs and achieve lasting improvements. The advantages of engaging stakeholders in model development processes have long been recognized; however, the methods for doing so are often not well-described.

**Objective:**

This paper aims to describe the participatory procedures that will be used to support systems modeling for national multisite implementation. The *Right Care, First Time, Where You Live* research program will focus on regional youth mental health applications of systems modeling in 8 different sites across Australia.

**Methods:**

The participatory model development approach involves an iterative process of engaging with a range of participants, including people with lived experience of mental health issues. Their knowledge of the local systems, pathways, and drivers is combined with the academic literature and data to populate the models and validate their structure. The process centers around 3 workshops where participants interact and actively engage in group model-building activities to define, refine, and validate the systems models. This paper provides a detailed blueprint for the implementation of this process for mental health applications.

**Results:**

The participatory modeling methods described in this paper will be implemented at 2 sites per year from 2022 to 2025. The 8 selected sites have been chosen to capture variations in important factors, including determinants of mental health issues and access to services. Site engagement commenced in August 2021, and the first modeling workshops are scheduled to commence in February 2022.

**Conclusions:**

Mental health system decision makers require tools to help navigate complex environments and leverage interdisciplinary problem-solving. Systems modeling can mobilize data from diverse sources to explore a range of scenarios, including the impact of interventions in different combinations and contexts. Involving stakeholders in the model development process ensures that the model findings are context-relevant and fit-for-purpose to inform decision-making.

**International Registered Report Identifier (IRRID):**

PRR1-10.2196/32988

## Introduction

Globally, it is estimated that more than 700,000 people lose their life to suicide each year, and suicide is the fourth leading cause of death among young people aged 15 to 29 years [1]. In Australia, suicide is the leading cause of death among people aged 15 to 49 years [2,3]. An estimated 340,000 (15%) Australians aged 18 to 24 years experience high or very levels of psychological distress [4]. Most mental health issues experienced during adulthood begin during childhood or adolescence and can result in disengagement from education and reduced social connections. This can, in turn, lead to reduced employment opportunities and poorer socioeconomic outcomes [5]. A recent national inquiry into mental health in Australia made recommendations that called upon multiple sectors to take action to be responsive to the needs of, and improve outcomes and experiences for, people with mental health conditions. The sectors identified by the inquiry went far beyond those usually associated with mental health services and included primary, secondary, tertiary, and vocational education; mainstream health services; early childhood education and care; disability support; workplace health and safety; finance; housing; insurance; justice and law; and digital health sectors [5]. Putting this broad context together with the individual variation in the etiology and course of mental ill health, deciding where and how to invest resources is difficult [6]. This challenge has traditionally resulted in investment in a *comprehensive and all-encompassing* package of interventions in which individual programs are often not sufficiently scaled and resourced to deliver impacts [7]. More effective decision support methods are needed to ensure that investment is well-targeted, coordinated, and implemented at a sufficient scale to deliver real impacts [7,8].

Governments have long been under pressure to provide more programs and services within constrained resources [9]. Policy and program decision processes are often complex, contextually dependent, and influenced by a range of competing priorities [10]. Decision support methods and tools are needed that can not only provide insights into which interventions or programs work but also facilitate identification of the context in which they work or whether other interventions, or combinations of interventions, will work better [9,11]. Systems modeling has a long history of providing decision support capability in a range of disciplines [12-14], including engineering, manufacturing, defense, business, and environmental sciences. It is increasingly being used for health applications. Systems models can be developed and used to leverage a range of data and evidence sources by combining them with local contextual knowledge and expertise to inform mental health system investment planning [8]. Involving decision makers and other stakeholders in participatory model development processes can increase the validity, credibility, and utility of models, ensuring they remain focused on priority policy questions and accelerating the mobilization of model insights into practice [11,15,16].

### Participatory Systems Modeling

The process of participatory systems modeling involves engaging multidisciplinary stakeholders in group model-building processes [17]. It can be used in conjunction with multiple modeling methods, including system dynamics, discrete event simulation, and agent-based modeling [18-21]. Various terms have been used to describe these activities, including participatory modeling, group model building, companion modeling (ComMod), and participatory simulation [21]. In the participatory modeling process, participants coconceptualize a problem and use modeling to describe and quantify the problem; identify, develop, and test potential solutions; and inform the decision-making and actions of the group [22]. For the purpose of this study, the term *participatory systems modeling* has been adopted and is defined as “a purposeful learning process for action that engages the implicit and explicit knowledge of stakeholders to create formalized and shared representations of reality [22] using computer simulation.”

The terms *stakeholders* and *participants* are both used in this study. By *stakeholders*, we refer to all who have a *stake* or involvement in the system [22], and the term *participants* is used to refer to those people who engage in the participatory modeling process. Therefore, the term *stakeholders* refers to a broader group of people.

Participatory systems modeling focuses on collaborative learning, and the tools and methods used in these programs promote system understanding and awareness among all stakeholders. The tools and methods used in participatory approaches may differ; however, the underlying principles are very similar and subscribe to the same basic aim—to actively engage end users and other stakeholders in model development to increase the robustness, validity, utility, and credibility of the models and facilitate their use to support decision-making processes [18,19,21,23-25]. Participatory modeling has been an important method in system dynamics modeling since its inception [18] and has been widely adopted in environmental modeling projects [19,23,26-29]. The advantages of engaging stakeholders in model development processes include the following [11,16,24,30]:

1. The contribution of extensive domain expertise of participants to model development
2. Social learning between participants and throughout the model development process
3. Joint problem framing to ensure that the model is focused on priority policy questions
4. Production of regionally customized and socially robust solutions, that is, solutions that are more likely to be trusted and accepted by decision makers and stakeholders
5. Identification and prioritization of evidence gaps
6. Identification of opportunities to insert the model into policy and program decision-making dialogues
7. Development of strategies to address communication challenges

Advances in modeling technologies have allowed greater model transparency and meaningful engagement in the model-building process by interdisciplinary groups [20,25]. Although modeling expertise is still required, modeling is no longer restricted to the computational and mathematical sciences and models are being designed to be broadly accessible across disciplines [31]. Participants engaged in the modeling process are able to inspect and critique the logic, parameters (values used), and assumptions of a model, and simulate scenarios independent of the modelers, using interactive model interfaces [25]. Broader access to, and engagement with, models can support faster model evolution and learning, particularly in identifying discrepancies between model results and empirical observations or knowledge concerning the world and helping to refine mental models across the group [14,19,32,33].

It may be difficult to understand and anticipate the impact of policy decisions on system behavior as a whole [12,14]. However, a quantified systems model can facilitate an increased understanding of system behavior by playing out the logical implications of introducing new policies and initiatives into complex systems, thereby making implicit assumptions explicit [25]. For example, in the mental health sector, participatory systems models co-developed with local health services have pointed to the importance of aftercare for people who have experienced suicidal behavior, a finding that is consistent across applications in diverse regions [34,35]. In addition, modeled intervention effects that initially seem counterintuitive may point to unanticipated consequences. For example, evidence-based community mental health education and awareness raising programs can lead to demand exceeding service capacity, increasing waiting times and disengagement with services, and increasing mental health–related emergency department presentations [36].

Transparent models can help to connect knowledge across the breadth of a team, enhance their ability to identify areas where their knowledge falls short, uncover logical inconsistencies, and contribute to more robust and strategic decision-making [25]. From this perspective, the discovery of an inconsistency between what the model suggests in simulation scenarios and empirical data is an opportunity to facilitate learning and refine the model to improve its forecasting capability, thereby increasing its value as a long-term decision support asset [20,25,28].

### Principles of Participatory Systems Modeling

Frameworks, guidelines, and principles for participatory systems modeling have primarily been developed within the environmental sciences field where it has been widely acknowledged that sustainability issues involve social processes and stakeholder engagement is necessary to support effective action [21,24,37-39]. These have ranged from highly prescriptive scripts used for group model building often associated with system dynamics modeling [18,19,23,40-42] to more general guidelines and considerations [20,21,24,32,43].

Participatory systems modeling projects are diverse, and flexible principles guiding the conduct of participatory processes that are also easily modifiable and applicable across sectors provide a practical approach to inform existing and future practices [21,24]. The following principles are described in Multimedia Appendix 1 and have been formulated based on recommendations from the literature [44] and the experience of the authors conducting participatory systems modeling for physical and mental health applications [16,17,30,34,45-49]:

1. Selecting and planning stakeholder engagement to ensure that appropriate expertise is available to guide model development
2. Being aware of social and group dynamics to facilitate inclusivity and give all participants the opportunity to contribute and having flexibility in the process to accommodate the priorities and preferences of participants
3. Maximizing transparency and openness in the model development process by ensuring that assumptions and data sources are made explicit
4. Iterating and refining by actively engaging participants throughout the model development process and incorporating their feedback
5. Encouraging learning and managing uncertainty through scenario analyses and hypothesis testing

Most strategic mental health policies and planning decisions are complex and not easily addressed using traditional analytic tools. Policy and planning decisions are challenged by multiple interacting factors with uncertain outcomes, competing options for action and investment, differing expert and local views of effective actions, and the potential for unintended consequences [17,50]. Scenario analysis can assist in identifying optimal combinations of interventions that remain effective even when the conditions in the system are varied [51]. An iterative, participatory approach to modeling allows the identification of data gaps and priorities for new data collection and development of ways to address these [11,20,21]. Models have significant potential to assist in good decision-making through the participatory process by bringing together best evidence, data, and knowledge and consolidating and testing shared hypotheses [25,32].

### Aims and Objectives of the Method Blueprint

Although the importance of involving stakeholders in model development processes to increase the relevance, validity, usability, and credibility of models has been recognized, the methods for doing so are not always well understood and terminology can be used loosely, leading to confusion. This paper aims to promote a shared understanding of our participatory systems modeling approach and provide a practical, detailed blueprint to support the implementation of the approach.

This paper was developed as a semistandardized guide for implementation across subnational regions as part of a broader program of participatory action research that aims to explore the feasibility, value, impact, and sustainability of building regional capacity in the use of more advanced decision support tools and technologies to inform systems strengthening and empower communities to address the mental health needs of young people [52,53]. The *Right Care, First Time, Where You Live* research program will use participatory methods to deliver contextually relevant systems models focusing on youth mental health services in multiple sites across Australia. More information about the research program is available [52]. The systems modeling program aims to provide regional health authorities, social service providers, and community stakeholders the tools, processes, and insights needed to more effectively allocate limited available resources and make compelling cases for further investments [53]. This paper outlines the participatory systems modeling procedures, methods, and activities that will be implemented to support this mental health multisite study related to youth.

The process described in this study builds on the experience of the authors conducting participatory systems modeling for a range of public health issues [6,8,16,17,30,34,45-48,54,55]. However, this study focuses on modeling for mental health applications in this multisite program. Operationalizing participatory systems modeling for mental health applications involves ensuring that the process is inclusive for people with lived experience of mental ill health and their support people.

## Methods

### Role Descriptions for the Interdisciplinary Core Project Team Members

The project roles mentioned later will be key to successful implementation. The descriptions are adapted from Atkinson et al [46] and Freebairn et al [16]. It should be noted that one person can play multiple roles in a project, that is, the project lead may also be the domain expert or the modeler may be experienced in modeling for policy so may also act as a translator. The term *primary partner agency* refers to the main stakeholder organization that will facilitate the modeling project in the local community. In the Australian health service context, this agency may be the Primary Health Network or jurisdictional health services.

A project lead facilitates the brokering and management of a project. This person will have the primary responsibility of engaging and maintaining relationships with participants and health service partners (ie, end users of the model). The project lead shares the duties of facilitating the modeling workshops with the lead domain expert and overseeing model development, associated documentation, and external communications.

A lead domain expert is a well-respected authority on the focus issue who can play a lead role in project planning and workshop facilitation.

A translator is a person who can contextualize the policy environment and data for the modeling team and translate the model requirements and development process to the participants.

Expert participants are people with a range of perspectives from across the system being modeled and policy, planning, and content area expertise, including representatives from local Aboriginal and Torres Strait Islander governance bodies, people with lived experience of mental health and suicidal behavior, representatives from federal and state governments, health and social policy agencies, local councils, nongovernment organizations, emergency services, research institutions, community groups, and primary care providers.

A dynamic simulation modeler is a person with expertise in systems modeling and ideally with a background in biostatistics, data science, or mathematics.

An economist is a person with expertise in economic evaluation of policy interventions, including multiple costing methodologies and different valuation techniques, with knowledge of decision analysis and priority-setting processes.

Superusers are nominated persons from within decision-making or primary partner agencies who will be socialized to the model and build competency in using it to explore policy scenarios and interpret and report model findings for reports, policy briefs, business cases, and advocacy.

Research or project support officers are responsible for coordinating the participatory systems modeling process, including logistical arrangements for workshops and liaising directly with the modeler and workshop participants to source and manage evidence and data requirements for the model-building process.

An expert technical adviser provides an independent review of the model, including model conceptualization, equations, and dimensional consistency, to identify errors and ensure that the model is robust and computationally efficient.

### Considerations When Including People With Lived Experience of Mental Health Issues and Their Support People

The advantages of involving people with lived experience of mental ill health, including carers, are well established and include ensuring that their essential knowledge about current care pathways, barriers, shortfalls, and what is needed from the mental health system, is embedded in research and service design to improve outcomes at individual, service, organization, and system levels [56-62]. The participatory modeling procedures described in this implementation protocol are informed by the literature regarding best practice principles for supporting consumer and carer participation in mental health research and as described by the National Mental Health Commission [56]. These procedures are also informed by experiences of the authors in implementing previous participatory modeling projects in the mental health sector in collaboration with people with lived experience of mental health issues and suicidal behavior.

The choice of location, venue, and timing for workshops should consider the needs of participants. Barriers to participation can include distance and travel times and environments that cannot accommodate individual requirements such as mobility access, space to take time-out breaks or dietary needs, and insufficient lead in time for workshops to allow participants to prepare for engagement, including taking a time-out from other commitments. Efforts will be made to provide an inclusive culture and safe environment at the workshops that supports the engagement of all participants [56]. In practical terms, this may include being explicit about the ground rules for safe and acceptable disclosure, ensuring that participants have access to and are aware of supports available, for example, debriefing and referral to professional support, allowing people to take a break when needed, and observing levels of psychological distress within the group; checking in with participants; and offering support where appropriate. People with lived experience may prefer to bring their peer support worker or carer to workshops as they can detect signs of distress well and can provide support as needed throughout the event, particularly when discussing suicidal behavior.

Workshop facilitators and support staff will ensure that large and small group discussions are respectful and inclusive for all participants, for example, valuing contributions from all participants, minimizing instances where people are interrupted or cut off or not listened to, and minimizing the use of jargon. Informal conversations during breaks and outside of workshops can be used to encourage participation in the process by providing additional information and clarification about the process and method and opportunities to contribute outside of the formal workshop process, for example, by sharing stories and experiences of the mental health system in direct, one-on-one conversation with the project team members.

The language used throughout the modeling process, but particularly during participatory workshops, will need to be inclusive and increase the likelihood that participants with lived experience of mental health issues feel respected and valued and able to contribute actively to the model development process. Language should be age-appropriate, respectful, nonjudgmental, jargon-free, and accessible to lay people. In addition, the language should be person-centered, that is, *person with mental health condition* rather than *they are mentally ill*, and recovery-oriented—conveying the potential for hope and opportunity. The Mental Health Coordinating Council provides an extensive and practical guide for using recovery-oriented language [63].

Ongoing evaluation of the process using participatory action research will be undertaken as part of the broader program to facilitate the identification of opportunities to improve consumer and carer engagement in participatory system modeling processes.

### Procedures for Building and Using Systems Models With Stakeholders

#### Overview

**Figure 1:**
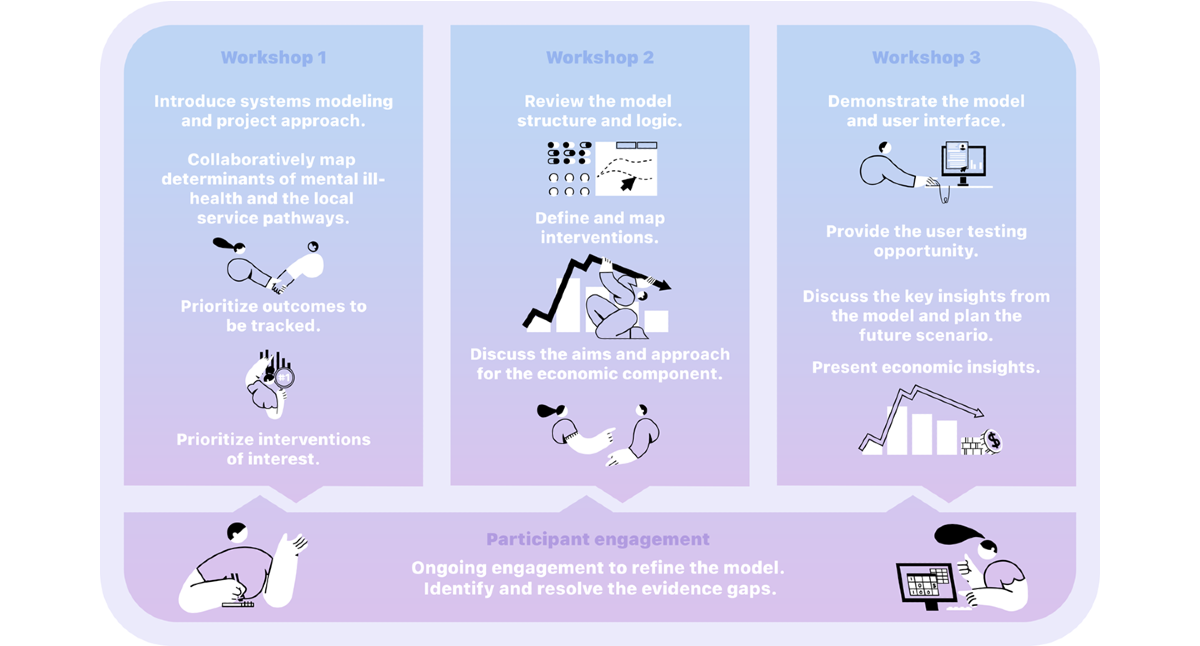
Overview of the activities involved in participatory modeling workshops.

Participatory model development involves an iterative process of understanding local systems, pathways, and drivers by engaging with local stakeholders and the academic literature, populating the model with data, validating its structure and performance with historic data, and ensuring face validity with stakeholders. It centers around 3 workshops where participants interact and actively engage in group model-building activities (Figure 1). Experts and key participants with an important *stake* in the topic are identified and invited to participate in the model development group (participants). Their expert knowledge and local practice experience are triangulated with research evidence and primary and secondary or administrative data sources to articulate the causal mechanisms driving mental health and suicide outcomes in a region.

Script-based approaches [18,19,41,42,64,65] have traditionally been used in system dynamics modeling. Our methods favor more organic, minimally structured activities that allow people to tell their stories and share their experiences, which elicits valuable systems information without the constraint of conforming to closely scripted activities or disciplinary conventions of systems science. This process aims to elicit information about what really happens on the ground, hidden incentives or disincentives embedded in the system, and information that is not available in academic papers or data sets but has significant implications for key outcomes and system functioning locally. Providing opportunities for interaction between participants as a group is valuable, as it often reveals more about the system than if the project team has met with participants individually. System learning also occurs when people hear the perspectives and challenges of those in other parts of the system who they may not frequently interact with.

The following sections describe the process for conducting participatory systems modeling workshops for mental health applications. It should be noted that this paper provides guidance specific to facilitating participatory systems modeling; however, it does not cover more general activities involved in workshop organization, for example, booking venues, arranging travel, organizing catering, managing RSVPs, and registration of participants. This is assumed knowledge for this paper.

#### Early Stakeholder Engagement and Workshop Preparation

Establishing effective partnerships is key to the successful implementation of participatory systems modeling projects. Early engagement with the primary partner agency for each site is important to ensure that the modeling will address their decision analysis needs. In some regions, improving mental health outcomes and reducing suicidal behavior will need to prioritize decision-making around investments to address youth justice, substance misuse, and unemployment challenges, where others may need to prioritize improving access to services. These discussions help to scope the model without changing the primary research question and the overarching purpose of the work.

There are four key activities in the engagement process: (1) determining the capacity of the stakeholder community to participate and actively engage in the activities; (2) communicating the purpose and goals of the systems modeling research and gaining commitment from stakeholders; (3) initiating engagement with the community and understanding current issues, challenges, and alternative perspectives; and (4) establishing relationships and building trust between researchers and community stakeholders [66,67]. The site and stakeholders engagement process to be implemented across sites will be undertaken using participatory action research principles.

Important considerations for this phase of the project include the following:

1. Identification of decision-making priorities for modeling: participating in a systems modeling process requires significant time investment for stakeholders. Motivation for stakeholders to participate is higher when addressing the mental health challenge is a high priority for the region, where the policy and planning environment is complex, where there are contested potential solutions, or where previous attempts at addressing the issue have not delivered impacts [16]. It is important to invest time in discussing local needs to ensure that the modeling is focused on answering the priority questions of the stakeholder group.
2. Identification and engagement of key contacts within the primary partner agency: once a key contact has been identified and engaged, their assistance will be sought to facilitate the identification and engagement of key stakeholders and organizations within their local community for youth mental health services.
3. Venue selection: there are two main considerations for selecting a venue for participatory workshops—first, the venue needs to have a facility to project presentations (including presentations of model architecture) onto a large screen where small text remains legible to participants. Second, the room needs to provide sufficient space for the participants to easily move around a large conference table. An important activity for workshop 1 is for participants to interact and engage in the conceptual mapping of the system. This involves the participants working with paper, tape, and sticky notes to contribute to the system diagram that is laid out on a large table.

At least 2 members of the project team will facilitate the workshops and at least another 2 will provide workshop support. Wherever possible, the workshop facilitators should include a local domain expert who works together with the modeler and project lead to facilitate the workshop. For example, in workshop 1, the local domain expert presents an overview of the epidemiology of mental health and suicidal behavior of the region being modeled and the modeler presents an introduction to systems modeling. The workshop facilitators jointly explain and support participatory activities. The workshop support staff provide logistic assistance to ensure smooth operation of the workshop. This includes welcoming and registering participants; ensuring that any necessary paperwork, for example, regarding participation, photography consent, and confidentiality have been completed; liaising with the venue staff; taking photographic records; ensuring that audio recording devices are in place and turned on during the appropriate sessions; setting out materials; and rearranging the room as needed to facilitate workshop activities. A list of materials for the core workshop activities is provided in Multimedia Appendix 1. It is beneficial, when there is sufficient capacity, for the workshop support staff to be workshop observers, recording field notes that can be used to support the project team debriefing and reflective analysis of each workshop. Field notes and observations would focus on levels of participant engagement in workshop sessions and questions and issues raised by participants during discussions and interactive activities. Runsheets may be developed for each workshop to clearly outline the roles of presenters and workshop facilitators and describe the activities and timing of each session in the workshop.

#### Participant Selection and Recruitment

Participant selection will be conducted in collaboration with the primary partner agency for each site to embed contextual knowledge into the participant selection process.

Purposive sampling will be used to recruit participants with diverse perspectives and expertise, including young people with lived experience of mental health issues, carers, members of the local Aboriginal and Torres Strait Islander community, mental health professionals, educators, policy makers, service planners, primary health care providers, health service managers, and other service providers. A comprehensive list of stakeholder categories is provided in Textbox 1.

**Categories of stakeholders to be considered for inclusion in the participant group.**

**Stakeholder categories**

- Health department policy makers and policy officers
- Local health district representatives
- Youth mental health researchers, including epidemiologists and social scientists
- Police and emergency services
- Clinicians from youth mental health and substance use support services
- City Council members and staff
- Primary care, general practitioners, nurse managers, and allied health professionals
- Educators, education department representatives, and school counselors
- Child protection workers
- Relevant nongovernment organizations and foundations
- Consumers, people with lived experience, and carers
- Community leaders, including church, traditional healers, and other leaders
- Health insurers and private service providers
- Hospital and other service administrators
- Program planners and service coordinators
- Call centers and web-based service providers
- Representatives from special interest groups, including lesbian, gay, bisexual, trans or transgender, queer, intersex and other sexuality, gender and bodily diverse people; indigenous; culturally and linguistically diverse; refugee
- Mental health promotion agencies

The aim is to include participants who are recognized as local leaders in consumer and carer experience, providing services, planning, and commissioning services and developing policies and the local Aboriginal and Torres Strait Islander community.

Selected participants will be provided with information relating to the aims of the project, the time commitment required, the likely timing of the workshops, and background information regarding systems modeling. Where possible, the initial invitation of participants is extended by the local domain expert or primary partner agency.

Before the first workshop, participants will be asked to provide written consent to participate, be recorded and photographed at workshops, and agree to not disclose confidential information shared by other participants during the model development process, which is important for providing a safe environment for sharing information that is important for model development but which the participants may not wish to be shared publicly.

### Workshop 1: Introduction to Participatory Systems Modeling and Conceptually Mapping the System

#### Overview

The logistics involved in planning and implementing workshop 1 and the activities undertaken to achieve the workshop objectives are discussed in detail. Although the overall aim of the participatory workshops is to maximize the active engagement and interaction of participants, it is necessary to initially present some background and context setting information. Therefore, once the welcome and introductory activities are complete, the initial sessions in the workshop involve presentations of relevant information to support the participatory systems modeling process. An example agenda is included in Multimedia Appendix 1. The five main objectives for workshop 1 are as follows:

1. Present an overview of the epidemiology of mental health issues and suicidal behavior relevant to the region or population catchment being modeled
2. Introduce systems modeling to the participant group to facilitate meaningful engagement in the model development process
3. Jointly conceptualize and map *system* structure and drivers
4. Prioritize the interventions and outcomes to be explored by the model
5. Introduce the economic analysis and explain that it will be developed following workshop 1, as the model’s purpose and scope develop

#### Welcome and Overview of the Project Aims and Objectives

It is important that participants are welcomed by the host organization in accordance with local customs, for example, in Australia, this would include an acknowledgment of the traditional owners of the country upon which the workshop is taking place and would also include an acknowledgment of the lived experience of mental health issues and recovery.

The aims and objectives of the project, drawn from project scoping discussions with the key stakeholder organization, will be presented to the participants. The overall aim may be quite broad, for example, “this project aims to use a co-designed systems model to provide a robust, evidence-based, interactive decision support tool to improve population mental health” or it may be quite specific “...a decision support tool to inform suicide prevention in young people,” depending on priorities identified in scoping discussions.

#### Session 1: Introduction to Dynamic Simulation Modeling (Time Allowed 30 Minutes; Purpose of the Session and Method)

The purpose of this session is to provide participants with an overview of what systems modeling is and what it can offer in a decision-making context.

A slide-supported presentation covering the points mentioned in Textbox 2.

**Method for session 1.**

**Method for session 1**

**Figure 2:**
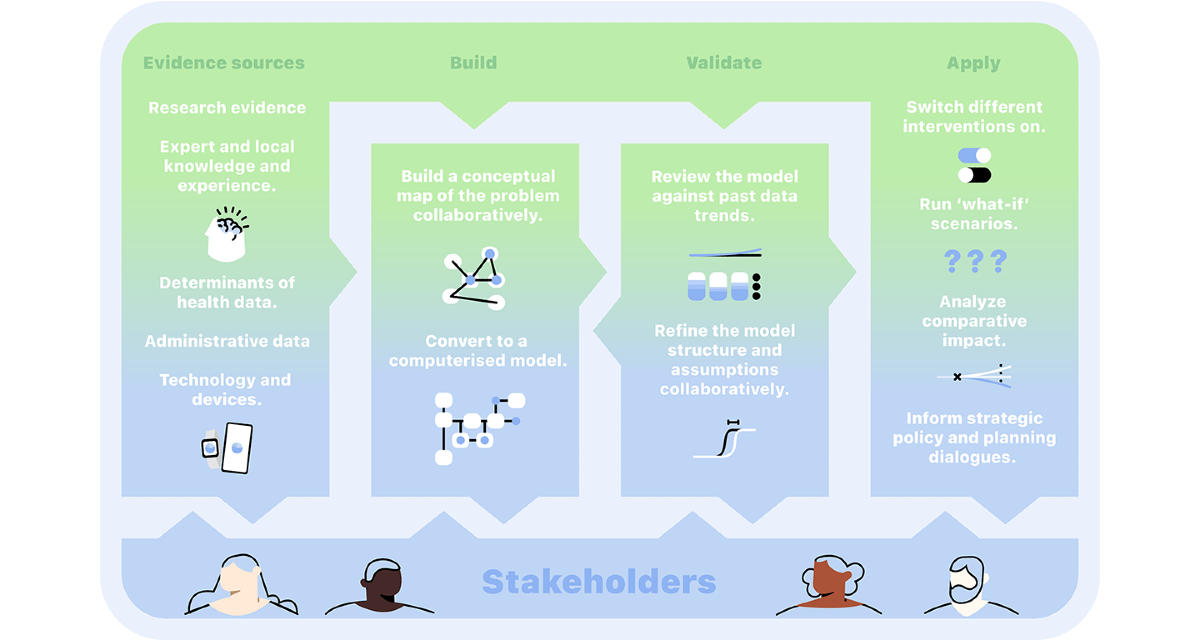
Overview of the process for building systems models using participatory methods.

**Figure 3:**
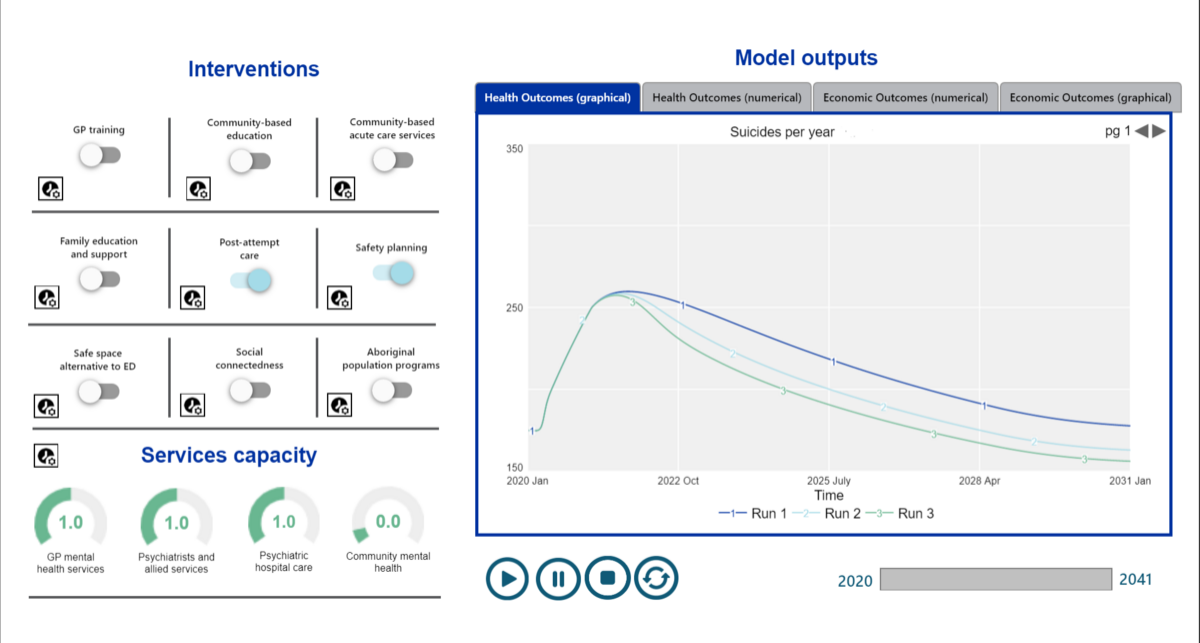
Example of the user interface for a suicide prevention model.

- Challenges faced in policy making and planning in the mental health sector:
  Complexity of problems, including complex determinants of mental health and suicidal behavior, population dynamics, and service pathways
  Broad range of options for intervening
  Changing mental health care and suicide support needs and demands over time
  Different perspectives and competing views of how best to intervene
- What systems modeling has to offer in mental health policy and planning:
  Explanation of what systems modeling is, that is, a simplified representation of the real world that can provide us with a method to map and quantify complex problems and service systems by bringing together a range of evidence, data, and knowledge. The developed model will be an interactive what-if tool for scenario analysis.
- The process for building a model and examples of a final *what-if* decision support tool:
  - Provide an overview of the process using a diagram such as Figure 2.
  - Emphasize the important role that participants have in this process. The participatory process is critical to understanding the behavior of the system and its drivers, identifying and considering potential unintended side effects of interventions, and keeping sight of the impacts of mental health initiatives on the wider health and social systems. By working together, the participant group ensures that the model is fit-for-purpose and can capture regional differences in demographics and service structures, making it a robust, contextually relevant decision support tool that can be embedded in the local policy or planning cycle.
  - Present example user interfaces (Figure 3) to demonstrate the outcomes of the project. Briefly explain the elements of the interface and how it can be used to explore the what-if scenarios.

#### Session 2: Introduction to System Dynamics Modeling (Time Allowed 30 Minutes; Purpose of the Session and Method)

The purpose of this session is to provide participants with sufficient knowledge of the basic concepts and graphical language underpinning system dynamics modeling to enable them to participate meaningfully in the model development process, including in the conceptual mapping activity in session 4, and the ability to critique the model in workshop 2.

The examples used here apply to system dynamics modeling; however, the introduction should focus on whichever modeling method is being used in the project, for example, state charts for agent-based modeling or process diagrams for discrete event simulation.

**Figure 4:**
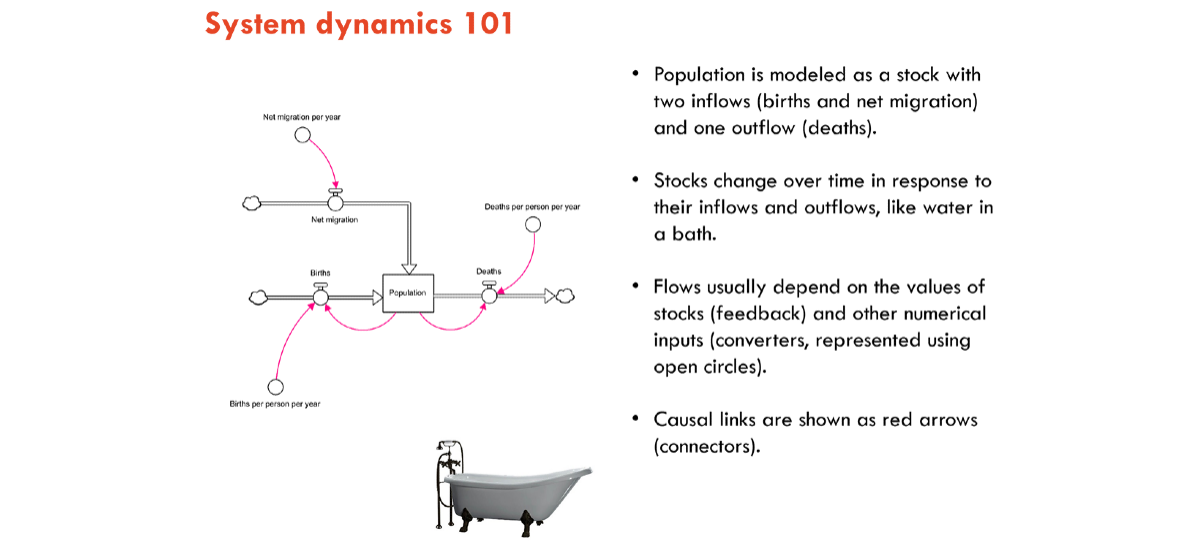
Facilitation slide to support the explanation of system dynamics concepts to the participants.

A slide-supported presentation covering the key terms and concepts of system dynamics modeling using visual diagrams (Figure 4) and plain language. The core concepts explained include the following:

1. Stocks and accumulations
2. Flows
3. Reinforcing and balancing feedback loops
4. How data will be used to calibrate the model

#### Session 3: Defining the System and Outcomes of Interest (Time Allowed 30 Minutes; Purpose of the Session and Method)

The purpose of this session is to provide participants with an overview of the mental health system and suicidal behavior in the local context and initiate discussions and priority outcomes to be tracked in the model.

It is preferable for the overview to be presented by a local, trusted domain expert and would cover the topics listed in Textbox 3.

**Method for session 3.**

**Method for session 3**

- Mental health outcomes, drivers, and service use
  - The focus of the models being developed in this program is on the complex interplay of social determinants, service system factors, population demographics, and behavioral dynamics that drive population-level youth mental health outcomes:
  - Epidemiology of mental health issues, suicide and self-harm, and service use (eg, emergency department presentations, psychiatric hospitalizations, primary care service contacts) relevant to the region. This usually begins with a broad overview and then narrows the focus down to the local context.
  - Other social- or system-level contributing factors in the local context, for example, alcohol and other drug use, family violence, service access, or unemployment.
- Eliciting outcomes of interest: this activity will be conducted as a facilitated discussion in this session or an interactive activity, and both procedures are described later. Unless it is culturally or contextually inappropriate to do so, the discussion should be recorded to ensure that the project team is able to refer back to the important details that they may not pick up while they are facilitating the workshop. The appropriateness of recording will be discussed with key stakeholders, for example, elders of the local Aboriginal and Torres Strait Islander communities, during preworkshop engagement:
  Facilitated discussion—a discussion starter slide presenting potential outcomes of interest that will be the primary outputs of the model. Commonly modeled outcomes for mental health applications include prevalence of psychological distress, mental health–related emergency department presentations, self-harm hospitalizations, suicide deaths, emergency department presentations and hospital admissions for alcohol and other drug use, quality-adjusted life years related to mental disorders. Participants are asked to discuss whether these outcomes are important for planning purposes and whether other outcomes should be prioritized.
  Interactive activity (requires pens, sticky notes, and a wall space):
    - Display a slide with potential outcomes to be measured in the model.
    - Ask people to write their top 3 priority outcomes (which can be different from those on the slide) on separate sticky notes and then put them up on the wall during a break. Participants are asked to place the notes in theme groups (ie, put their sticky notes together with other similar notes).
    - The modeling support team provides feedback about the outcomes of interest in session 5 (detailed later). Feedback would include ranking the prioritized outcomes (ie, identifying the outcomes that were nominated most frequently by participants) and grouping the identified outcomes into broad themes. Further analysis to ensure that the main outcome themes identified by the participants have been captured can be undertaken after the workshop. Where there is disagreement among the participants about which outcomes should be prioritized, a voting process will be used to resolve it.

#### Session 4: Participatory Mapping Exercise (Time Allowed 1.5-2 hours With a Break Partway Through; Purpose of the Session and Method)

The activity undertaken in this session provides the most critical outcome for workshop 1, a co-designed conceptual map of the system to be modeled. In this activity, participants interact with each other and the project team to jointly conceptualize and qualitatively map the youth mental health system in the form of a *draft model structure*.

The mapping exercise is undertaken using the procedures listed in Textbox 4.

**Method for session 4.**

**Method for session 4**

**Figure 5:**
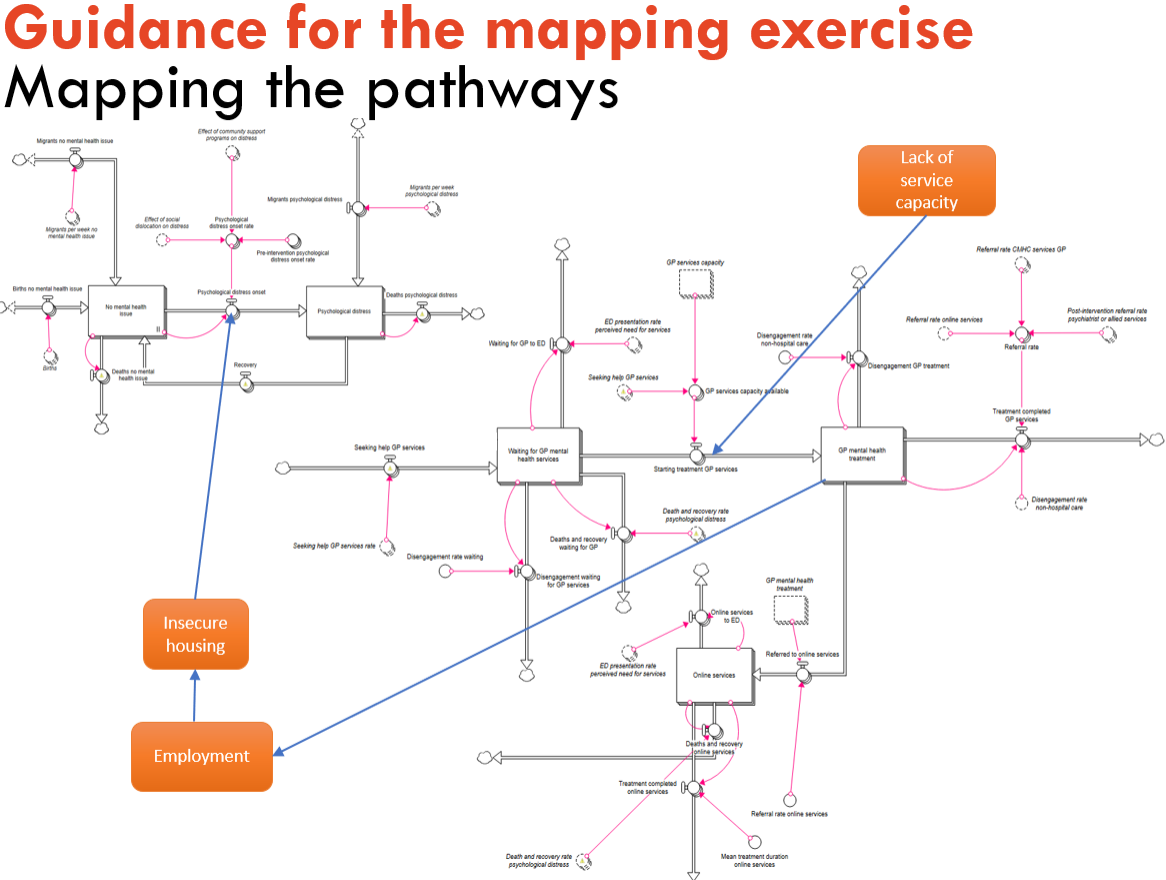
Facilitation slide to guide the conceptual mapping activity. GP: general practitioner.

- A simple draft model structure that incorporates key stock and flow structures for system dynamics models (or state charts for agent-based models) is derived based on preworkshop engagement with stakeholders and on previously published mental health systems modeling research in a similar context [48]. The structures might include aging chain structures and stock and flow diagrams representing the change in psychological distress over time or changes in demand for services and service pathways.
- The structural components, such as the stock and flow structures identified earlier, are preprinted, laminated, and laid out on large sheets of paper in preparation for the workshop. This provides a *straw man* structure for participants to modify and elaborate on and a starting point for discussion.
- The guiding instructions explain to participants that a draft model architecture has been laid out to provide a starting point for the mapping activity and that they are invited to review, contribute to, and improve the map. For system dynamics modeling projects, participants are asked to focus on high (or system) level factors that influence the *flows*, for example, community- or service-level characteristics rather than individual behavior or choices. Participants are encouraged to expand the structure to capture the key service pathways of the region.
  Instructions should emphasize the following:
    - This *brainstorming*-style activity aims to map the important causal pathways that contribute to the focus issue, that is, youth mental health issues.
    - The mapping activity uses elements of the model structure presented in session 2.
    - It is an inclusive process that demonstrates the capacity for systems thinking to help understand mental health issues, suicidal and self-harm behaviors, and their determinants. The objective is to understand what pathways are missing, what influences the flows, and how changes in one part of the system impact other parts.
    - Present slides showing a draft model architecture that participants can expand and critique. The draft is intended to initiate discussion rather than impose a pre-empted model design. Figure 5 illustrates an example slide showing a draft stock and a flow diagram. The slide is used to explain how factors that influence the flows between the stocks can be mapped to the diagram and that the direction of influence is important. Instructions will be tailored for each site by using real-world examples derived from preworkshop engagement discussions.
    - Advise participants that the activity will be photographed and audio-recorded to ensure that the project team can incorporate their contributions into the model accurately.
  The project team should circulate among participants during the activity to answer questions and engage in discussions. Participants will often verbalize where the map requires further development but may need encouragement to physically put down their thoughts on paper using the materials provided.
- Prompting questions are used to facilitate the activity. For example, the following general questions can be presented on a slide that is left on display during the activity, but the project team may develop more specific probing questions that can be used to facilitate small group or one-on-one discussions with participants during the activity:
  Have we captured the important stocks? Are there others that should be included?
  What factors influence the flow between stocks (family and environmental factors)?
  Are there other stock and flow structures that should be included?
  Are there any incentives in the system that influence behaviors or flows?
  Are there any feedback (positive or negative reinforcing cycles)?
- The project team uses the scheduled break time to debrief about the mapping activity and determine the important areas to focus on in the second part of the session, for example, “Are there other causal pathways, barriers, or disincentives in the system of interest that have not been elicited?” “Is there anything on the map that the team does not understand or need to clarify?” “Are there feedback loops emerging from the map that can be elicited more clearly?”
- Reconvene the activity by inviting participants to return to the conceptual map and continue to add any additional elements that they have thought of during the break. The project team circulates among the participants, encouraging them to make any last contributions to the map.
- Session 4 will finish with a large group discussion to do the following:
  Acknowledge participants’ valuable contribution to conceptually mapping the system.
  Refine, clarify, and define the elements of the model structure. This discussion would vary depending on the purpose of the model, but may include, for example, defining age ranges or priority subgroups of interest.
  Identify data sources and research evidence to inform the model for each prioritized subgroup. Participants are asked to complete the data contribution form.

#### Session 5: Prioritizing the Interventions of Interest (Time Allowed 45 Minutes; Purpose of the Session and Method)

The purpose of this session is for participants to identify the priority interventions of interest in modeling projects to ensure that the appropriate level of detail is included in the model structure to capture their effects.

This activity will either be run as a facilitated discussion and voting process conducted at the workshop or a larger community engagement process, including the facilitated discussion described later, and a postworkshop survey sent out via workshop participant networks where community members vote on which interventions are the highest priority for inclusion in the model. The decision regarding which selection method to use would be made in consultation with the primary partner agency to best meet the needs of the site. Regardless of the method used, diverse views are likely to be expressed. Both prioritization methods are described in subsequent sections. The voting or survey processes generally identify the priority interventions (or outcomes); however, this is followed up with a discussion in workshop 2 that aims to achieve a broader consensus. The workshop discussion option is described in Textbox 5 and the post-workshop survey is described later.

**Method for session 5.**

**Workshop Discussion Activity**

**Figure 6:**
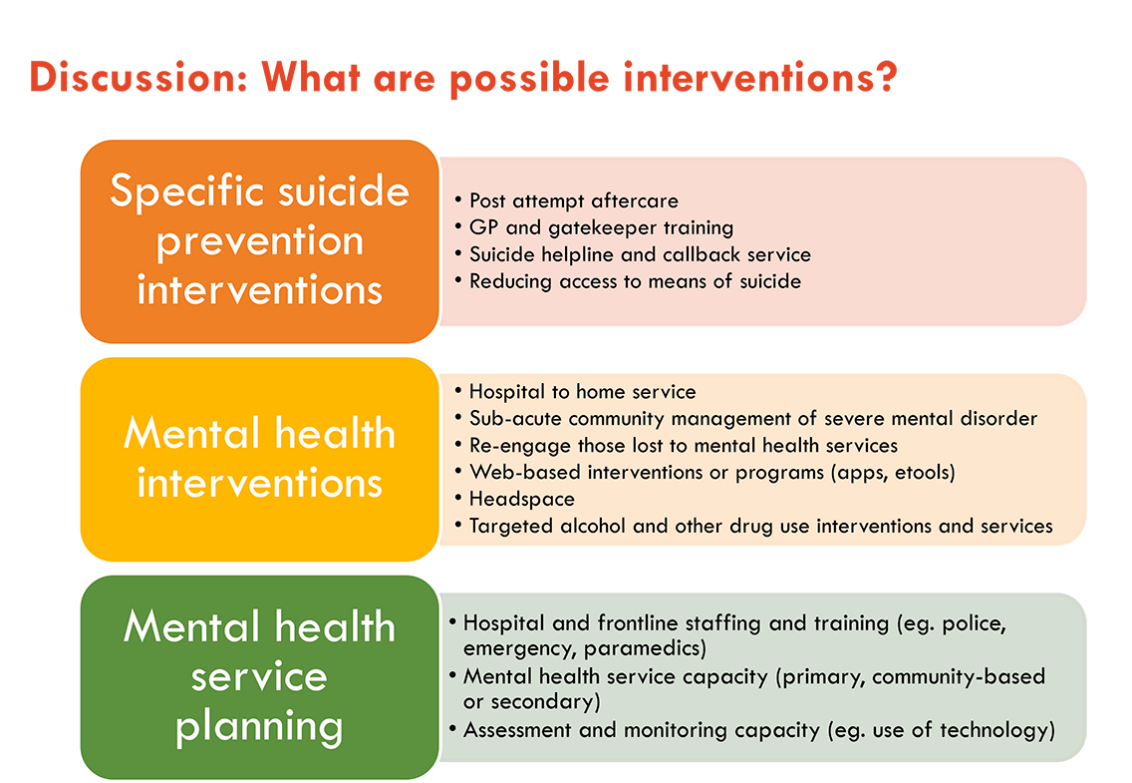
Example slide presenting possible interventions to be considered for inclusion. GP: general practitioner.

- Introduce the session, explaining the importance of understanding which interventions are of priority interest to the participant group to ensure that the model is built in a way that can adequately incorporate the intervention effects.
- Explain that the modeled interventions can be based on interventions already implemented in the real world or that they can be hypothetical interventions. Both are useful for *what-if* scenario testing. Interventions across the full spectrum of services can be considered for inclusion in the model, for example, primary prevention, early intervention, acute care, rehabilitation, and aftercare. Scenarios examining the impact of disinvestment in existing programs can also be explored and, if deemed a priority, should be identified at the outset. It is generally feasible to include 6 to 8 interventions in a participatory system dynamics modeling project that is 6 months in duration.
- Present a slide showing a list of potential interventions derived from the preworkshop engagement discussions (Figure 6). Explain that the focus of the current session is to prioritize and define the interventions to be integrated into the model:
  Ask participants to write down their priority interventions on separate sticky notes (which may or may not include those in the presented list).
  The participants will place the notes on a wall surface and group their own with similar interventions placed by other participants. Advise participants that we are interested in a range of interventions, but it does not matter if someone else writes down the same one.
  The participants are given 10 sticky dots to allocate to their interventions of choice. They can place as many dots as they wish on their priority intervention. More dots placed against an intervention emphasizes its importance.
  The project team groups the interventions into themes and records the voting results following the workshop. The results are presented in workshop 2.

##### Postworkshop Survey Activity to Facilitate Wider Community Consultation

When broader community consultation to prioritize interventions is preferred by the primary partner organization, it is facilitated using the following procedures:

1. Follow the aforementioned steps outlined in the first two bullet points in Textbox 5.
2. Present a slide showing a list of potential interventions that have been derived from the preworkshop engagement discussions (Figure 6).
3. Explain to the participants that their support will be required to distribute a survey to the wider community, asking people to prioritize which interventions are most important, from their perspective, to be included in the modeling process. Explain that it will be important for the participants to encourage people in their network to complete the survey to ensure that the voice of the community is heard in the model-building process.
4. Describe the survey process, for example, how the survey will be distributed and the timeframe for responses, and that the results from the survey will be combined with the workshop discussions and presented back to the participants in workshop 2 as a prioritized list of interventions.

#### Session 6: Economics, Next Steps, and Data Contribution (Time Allowed 30 Minutes; Purpose of the Session and Method)

There are two purposes for this session as follows:

1. To introduce the economic approach that will be used and explain the data requirements for the analysis.
2. To highlight the progress and valuable contributions made in workshop 1, discuss potential timing for workshop 2, identify sources of data and evidence to inform the model, and invite participants to contribute their expertise outside the workshop process.

Facilitated discussion supported by slide presentation:

1. Economics lead introduces the role of economic analysis in the project and how it can be used to contribute to the decision support purpose of the modeling process. A broad overview of the economic approach is provided, including an explanation of the types of economic analyses that may be used and how this can be guided by model purpose and available data.
2. Facilitators present a summary of the achievements over the course of the day, for example, applying systems approaches to understanding the mental health system and suicide prevention, including collaboratively mapping the contributing factors and service system. Link these achievements back to the overall project aims and objectives by explaining that this is the start of the process to develop a decision support tool.
3. Revisit the participatory process, explaining the activities in each of the 3 workshops and engagement that will happen between the workshops.
4. Propose approximate timing for workshops 2 and 3 and encourage participants to identify how they can play an active role in the development of the model by indicating to the project team any data sources that may be of use for model development and their willingness to be contacted out of session.

#### Concluding Session (Time Allowed 10 Minutes)

This session is an opportunity for a representative from the hosting stakeholder organization to thank the participants for contributing their time and expertise to model development.

Allow the opportunity for any participants to contribute concluding remarks either in a group format or individually to the project team.

### Workshop 2: Defining, Refining, and Mapping Interventions

The main objectives of workshop 2 are to provide an update on progress since workshop 1, present the current version of the systems model to the participant group, jointly conceptualize and map the interventions to be explored in the model, refine the outcomes to be measured, and outline the health economic components of the project in detail. The workshop sessions and activities undertaken to achieve these objectives are discussed in detail later, and an example agenda is provided in Multimedia Appendix 1.

#### Welcome Back and Recap From Workshop 1: Purpose of the Session and Method

The purpose of this session is to reintroduce participants to each other, the project, and the methodology.

A facilitated discussion and slide-supported presentation covering:

1. Welcome back and housekeeping
2. Recap of workshop 1—a short presentation recapping the overall project aim, the activities, and outcomes from workshop 1; the consultation and interactions that have taken place since workshop 1; and the project timeline
3. Aims of this workshop—a brief overview of the purpose of and activities planned for this workshop

#### Session 1: Presentation of the System Dynamics Model (Time Allowed 60 Minutes; Purpose of the Session and Method)

There are 3 purposes for this session as follows:

1. To present the current draft version of the model structure and logic.
2. To ensure that the model is transparent and familiar to participants.
3. To elicit feedback from participants on the model structure, logic, and data used to parameterize or calibrate the model.

**Figure 7:**
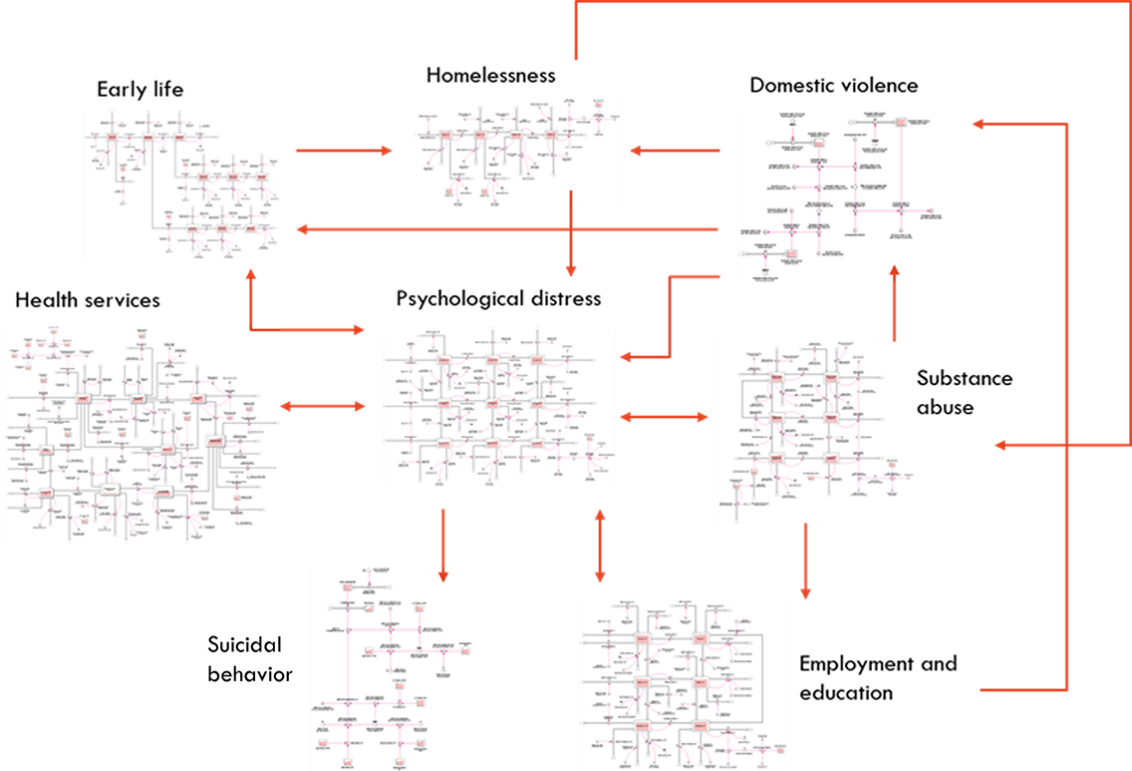
Example model overview showing the main components of the model structure and how they relate.

A slide-supported presentation by the lead modeler with facilitated discussion:

- Recap the building blocks of system dynamics, explaining stock and flow diagrams and model initialization values and parameters, and then demonstrate how they are combined to build a representation of a complex mental health system (see the example slide in Figure 5 from workshop 1).
- An overview diagram of the model is presented, showing the main components of the model and how they fit together (Figure 7). This can be emphasized using examples of within-component and between-component dynamics [48]:

...for example, within the health system component, the proportion of the population waiting for services, receiving services, or disengaging from services changes over time based on service system capacity and the rates of flow into, within, and out of the service system. Dynamics also occur between the model components, for example, as unemployment rises, not only does it directly act to increase the incidence of high to very high psychological distress in the modeled population (which has flow-on effects on rates of substance misuse, and adverse early life exposures), but it also increases rates of domestic violence and homelessness, both of which further increase the rate of psychological distress.

- The modeler explains the logic and structure of each model component. Example diagrams of model components, for example, the psychological distress component, are available elsewhere [48]. Uncertain parameter estimates and assumptions needing clarification for each component will be identified and discussed with the participant group in this session. Where available, it can be useful to include outcomes of the calibration process, demonstrating how well the model fits historical data trends which build confidence in the model’s causal hypothesis. Participants are encouraged to probe, ask questions, and provide further information and feedback that will assist in refining the model.

#### Session 2: Intervention Mapping (Time Allowed 30 Minutes; Purpose of the Session and Method)

The purpose of this session is to provide feedback to participants about the interventions prioritized for inclusion in the model.

A slide-supported presentation by the project lead and local cofacilitator with facilitated discussion:

1. Present results from the previously conducted intervention prioritization process. Two options have been proposed in this study to elicit the intervention priorities. The first option involves an activity in workshop 1 where participants nominate and vote on interventions to assign priority. The second option involves distributing a survey through participants’ networks to gauge community perceptions of which interventions are the highest priority to model. The results of the chosen process are represented in this session.
2. Include a slide with other intervention options that have been identified or discussed after workshop 1 but were not included in the original list, if appropriate.
3. Discuss with the participants whether they still agree with the prioritized list of interventions or modifications need to be made. This is a facilitated discussion to achieve a broad agreement on the suite of interventions to be modeled. However, where very strongly held views remain about the inclusion of an intervention or outcome that is not prioritized, the modeling team attempts to accommodate this if possible or note it for later development of the model.

#### Session 3: Intervention Mapping Exercise (Time Allowed 1.5-2 Hours; Purpose of the Session and Method)

The activity undertaken in this session provides the most important outcome for workshop 2, mapping intervention effects to the model structure. In this activity, participants interact with each other and the project team to jointly define the prioritized interventions and identify where they are likely to have their effect in the model structure.

A facilitated activity conducted in small groups according to the procedures listed in Textbox 6.

The preprinted copies of the model structure are provided to each of the small groups to map the mechanism of the effect of the intervention directly to the model structure. Different color markers can be used for each intervention on a single printout, or multiple printouts can be used, one for each intervention. The important factor is ensuring that each intervention is clearly differentiated.

A member of the project team will work with each small group to assist them in working through the questions and the mapping activity and respond to any questions raised.

It can be useful to split the intervention mapping work over 2 sessions with a break in the middle to allow the project team to discuss whether there are gaps in the discussion, allow the participants to interact, or approach the project team for a one-on-one conversation to provide information that they would prefer not to share in a group discussion.

**Method for session 3 (intervention mapping exercise).**

**Method for session 3 (intervention mapping exercise)**

- Each group will focus on a set of similarly themed interventions. For example, interventions for suicide and self-harm, such as community-based crisis response teams, using technology by crisis response workers to facilitate assessments, and postattempt care and follow-up could all be considered and mapped by one group of participants.
- Participants self-select the group of interventions that they would prefer to work on.
- Introduce the activity to the participants by explaining that it involves two aspects. First, the intervention is defined and described by working through the questions given later (provide a printed sheet with questions for each intervention), and second, mapping is carried out to determine where in the core model structure the intervention is likely to have an effect. The following questions will be used to guide the small group work:
  Definition of intervention:
    - How would you define the intervention specifically?
    - What are its components?
    - Has the intervention been piloted or evaluated before?
  Mechanism of the effect:
    - Where does the intervention in the core model structure have its effect? Is there a particular variable on which the intervention acts, and what is the nature of this effect?
    - What levels of reach and adoption (uptake) would be considered reasonable targets for this intervention? What levels of reach and adoption are we currently achieving (if appropriate)?
    - Are there differences in the effectiveness of this intervention for the key population subgroups represented in the model?
    - Does the delivery mechanism have an impact on the effectiveness of the intervention?
    - Are there any particular data sources and research in this area that you know of that is essential for us to refer to?
  Consequences of the intervention:
    - Are there any unintended consequences or feedback loops (explain with relevant examples)?
    - How can the intervention be implemented (eg, phased or universal roll-out)? Are there any factors that would influence the implementation of the intervention (barriers and facilitators)?
    - What is a reasonable estimate for the amount of time it would take to scale up this intervention: 1 year, 2 years, or 5 years?
  Anything else?
    - Are there any other important issues or factors to consider when representing this intervention in the model?

#### Session 4: Economic Component (Time Allowed 30 Minutes; Purpose of the Session and Method)

The purpose of this session is to provide an overview of the aims and intended approach and encourage participant feedback.

A slide-supported presentation of approximately 25 minutes, with an additional 5 minutes of questions at the end (Textbox 7).

**Method for session 4 (economic component).**

**Method for session 4 (economic component)**

- Introduce the role of economics as equipping the model to enable it to undertake *dynamic priority setting and economic evaluation*. This will generate outputs to support business cases for investing in interventions, which may include the allocation of new budgets or disinvestment in programs that can be reinvested differently to improve population outcomes. Explain that the economic analysis will also support the moral case for interventions where outcomes are not monetized, and the focus is retained on the most efficient delivery models to avoid self-harm and suicide. Clarify that the process integrates economic information and valuation techniques into the model rather than conducting it as a separate exercise.
- Outline that there is a menu of different possible approaches and techniques to choose from and that this choice is conditional upon the model scope, purpose, and information needs of decision makers who would fund interventions (eg, health and non–health sectors may require different information).
- Summarize the key points from workshop 1 regarding the emerging scope and purpose of the model. This sets the context to explain the choice of economic approach and methods and how that is intended to be aligned with participant needs.
- Describe the three generic stages to the economic approach:
  Stage 1 involves estimating the financial and human cost of business as usual, including, at a minimum, quality of life and health service activity costs. Conditional upon model purpose and data availability, this can be widened to include nonhealth impacts, such as productivity and impact on carers.
  Stage 2 involves costing priority interventions, conditional upon sufficient detail, such as specific service delivery models. Explain that if interventions are not well defined, then they cannot be properly costed. In that event, the default approach can be using a what-if analysis that can estimate the potential impacts of introducing an aspirational intervention on flow-on costs and outcomes. This is intended to support the development and testing of specific service delivery models.
  Stage 3 involves making the value proposition to invest by describing how interventions can reduce the burden of continuing with business as usual.
- Explain that the economics will then ensure that the model can tailor the business case for investment to meet potentially different funder expectations and normative positions, such as (1) a return on investment (eg, invest to save), (2) cost-effectiveness (cost per health outcome and health utility unit), and (3) cost benefit (all costs and outcomes included, where possible, and valued in dollar terms). The rationale for this approach is to help foster action and, where necessary, encourage multisector approaches.

#### Session 5: Concluding Session (Time Allowed 15 Minutes; Purpose of the Session)

The purpose of this session is to acknowledge the valuable contribution made by the participants at the workshop and note the likely timing for workshop 3. This session provides an opportunity for a representative from the primary partner organization to thank the participants for their ongoing contribution to model development. This session is also an opportunity for the participants to contribute concluding remarks either in a group format or individually to the project team.

### Workshop 3: Introducing the User Interface and Delivering Model Insights

The main objectives of workshop 3 are to present the penultimate version of the systems model to the participants by walking them through a high-level summary of the model, highlighting any major changes since the previous workshop and the user interface and demonstrating how it can be used to simulate intervention scenarios and preliminary insights from the model. The activities undertaken to achieve these objectives are discussed in detail later, and an example agenda is provided in Multimedia Appendix 1.

#### Welcome Back and Progress Update: Purpose of the Session and Method

The purpose of this session is to reorientate participants to the project and methodology.

A facilitated discussion and slide-supported presentation covering:

1. Welcome back and housekeeping.
2. Recap of progress—a short presentation recapping the activities and outcomes from workshops 1 and 2, including presenting back the list of interventions that were prioritized at workshop 2 and the consultation and interactions that have taken place outside the workshop settings.
3. Aims of this workshop—a brief overview of the purpose of and activities planned for this workshop.

#### Session 1: Demonstration of the System Dynamics Model (Time Allowed 45-60 Minutes; Purpose of the Session and Method)

This session has 3 objectives as follows:

1. To represent the high-level model structure and logic.
2. To advise on any major changes based on feedback from workshop 2.
3. To demonstrate model use to participants.

A slide-supported presentation by the lead modeler with facilitated discussion:

1. An overview of the model will be presented, showing the main components of the model and how they fit together. An example of this is shown in Figure 7.
2. Updates or revisions to the model since the previous workshop will be described. The description will emphasize where participant feedback has been incorporated into the model.
3. Additional slides focusing on revised and newly added model components, for example, the structure for one or more example interventions, can be presented.
4. Demonstrate the use of the model interface, including running scenarios in the live model.
5. Run a set of intervention scenarios to draw out key model insights. The modeler is often required to explain the reason behind some insights, particularly if they are counterintuitive.
6. Discuss the policy and planning implications of the initial model insights.

#### Session 2: User Interaction With Model (Time Allowed 60 Minutes; Purpose of the Session and Method)

The purpose of this session is to provide an opportunity for participants to gain experience using the model to explore scenarios and provide feedback on the functionality of the user interface.

This is an interactive session in which the participants interact with the model interface. A member of the project team is stationed with each computer to provide guidance, interpretation of findings, or technical assistance, where required.

The project team should ensure that access to one computer per 5 to 7 participants is available so that a diverse group has the opportunity to interact directly with the model.

Feedback questions can be printed or presented on a slide to guide feedback on the model interface as the participants interact with it. The feedback questions can be tailored to ensure relevance to different modeling projects but, in general, would include the following:

- What should the available ranges be on the slides?
- Are there any labeling or language issues that we need to address?
- Any other comments on the interface or model?

Feedback can be given directly on printed screenshots of the model interface provided for each small group. This session will likely generate further questions about the model and discussion about the results of the simulated scenarios. The lead modeler will be available to move between groups, as necessary, to respond to technical questions and assist with interpretation. This small group discussion is an opportunity for the project team and local domain lead to engage with the participants to ascertain where further clarification is needed, for example, how to vary input values or how to interpret results of simulated scenarios.

As highlighted in the aforementioned feedback questions, it is very important to ensure that the language used in the model interface is accurate, understandable, relevant, and acceptable for end users. The highest priority is to ensure that the language used does not inadvertently alienate or offend participants. Ideally, the model interface will be *user-tested* with participants, for example, from the primary partner agency and other key user groups, such as reference groups for people with lived experience or Aboriginal and Torres Strait Islander communities, before being presented at the workshop.

#### Session 3: Health Economics (Time Allowed 30 Minutes; Purpose of the Session and Method)

The purpose of this session is to provide a recap on the approach taken and demonstration of key analyses and findings. If the model is not yet fully developed, then an update should be given with timelines for completion.

A slide-supported presentation of 25 minutes with 5 minutes for questions and clarifications:

1. Recap on the approach of the economics and how that is aligned with model purpose and participant needs following discussions at workshops 1 and 2. Then provide results (or updates) on the three stages of analysis.
2. Stage 1: establishing business as usual—estimating the financial and human cost.
  Provide a selection of activity-based service costs for exposition (such as hospitalizations). Describe the estimation process and how modeled populations acquire costs as they reside in service stocks to illustrate how the economics is layered into the model. Repeat for health utilities and explain how quality-adjusted life years are estimated. If relevant, continue the exposition for wider impacts, such productivity and carer impacts.
3. Stage 2: *costing interventions*. Provide the costing estimates for the priority set of interventions and explain how these were derived. Highlight if, and why, certain interventions could not be costed properly because of insufficiently defined service delivery models. Reiterate that a what-if analysis can be conducted.
4. Stage 3: *making the value proposition*—creating the business case and supporting the moral case. Provide an illustration by selecting 2 examples to demonstrate the value of investing in interventions to reduce distress. One example can select a single intervention (which could be a *what-if* analysis) and the other should be a combination of interventions to demonstrate the capability of the model to develop an optimal intervention portfolio relative to a budget. These examples may include one or a combination of valuation methods, namely, return on investment, cost-effectiveness (utility), and cost benefit.
5. Use screenshots from the economic component of the model dashboard for ease of exposition and demonstrate to participants how they can also use the controls in the dashboard to select interventions and generate economic outputs and how this can feed into a relevant business case for investment.

#### Session 4: Concluding Session: Next Steps, Closing Remarks, and Feedback

The workshop facilitators will describe the achievements from the project and the valuable contributions made by the participants. The facilitators will explain how the model will be made available to the participant group, how the model will be used to inform regional decision-making, how the model will be maintained, and how ongoing technical support will be provided. Ideally, additional modeling informed strategy dialogues are hosted by the primary partner agency to build a collaborative consensus for action through discussions with broader community stakeholders and interaction with the model. The project team will provide support as needed to that process, including superuser training to build capacity in the independent use of the model.

This session is also an opportunity for a representative from the hosting stakeholder organization to thank the participants for their contribution to the model development. It will also give opportunity for any participants to contribute concluding remarks either in a group format or individually to the project team.

In our approach, we invite all participants to let the project team know if they are interested in coauthoring peer-review publications from this study. This is an important recognition of the substantial time and intellectual contribution that the participants have made to the development of their local model. Participants who do not engage in coauthoring papers are, with their consent, acknowledged as members of the modeling consortium.

### Ethics

Systems modeling processes do not routinely require ethics approval as they involve secondary analysis of data and are considered a process of evidence synthesis. However, ethics approval was requested and granted by the Sydney Local Health District Human Research Ethics Committee (protocol number X21-0151 and 2021/ETH00553) for the participatory action evaluation research being conducted alongside the participatory systems modeling processes across the 8 sites.

## Results

This study has been developed for implementing participatory systems modeling in the *Right Care, First Time, Where You Live* program, which is funded from 2021 to 2025. The methods described in this paper will be implemented at 2 sites per year from 2022 to 2025. The 8 selected sites include urban, regional, and rural or remote mental health service settings and are chosen to capture variations in socioeconomic conditions and demographics, population density, mental health risk profile, and access to mental health care and other services. Initial site visits were conducted between August and December 2021.

## Discussion

As decision makers navigate complex policy environments, including mental health, there is a need to leverage interdisciplinary problem-solving and advanced decision support tools, such as systems modeling, to mobilize a wide range of evidence, data, and other forms of information to inform effective decision-making [14,32,33,68-70]. The involvement of stakeholders and coproduction of knowledge are critical to ensuring that model findings are policy-relevant and can be used to inform decision-making [11,16,25]. In the mental health context, decision makers must consider multisectoral determinants of mental health issues, regional variation and changing local population needs, competing views about *what works*, and restricted resources and workforce [6].

Systems modeling provides a quantitative method to combine the consideration of individual behavioral, social, cultural, economic, and service risk factors and captures the complex, nonlinear interrelationships, feedback loops, and threshold effects that characterize mental health systems [71]. Actively engaging stakeholders in the development of the systems model ensures transparency and increases their understanding of the model as a decision support tool, which, in turn, increases the likelihood of the model being embedded in policy and planning cycles to target investments more strategically in mental health programs and services [6,7,16]. This protocol describes a structured process that combines diverse perspectives and facilitates interdisciplinary dialogue in the development of systems models for mental health services, which can be adapted for other applications, topics, and settings. Importantly, the protocol includes considerations to ensure that people with lived experience of mental health issues and their supporters can meaningfully contribute to the process.

The participatory process detailed in this protocol emphasizes participant interaction and opportunities to draw out participant knowledge and expertise and actively involves participants in decision-making, giving them a voice and a stake in the outcome. The protocol builds on previous research that identified that the benefits of the participatory process include the enhancement of professional networks; increased transparency and, therefore, familiarity and trust in the model; integration of significant knowledge and evidence into the models; identification and facilitation of policy insights and opportunities to apply model findings in practice; and identification of key messages to deliver to a broader policy and practice audience in a way that is compelling and engaging [16,17,30].

Despite providing acknowledgment of the importance of including end-user stakeholders in model development, many participatory modeling projects do not explicitly describe or reflect on the participatory process component of the project [11,22,24]. This protocol responds to international interest in these methods and provides a blueprint for operationalizing participatory systems modeling based on years of applied systems modeling research for mental health and broader public health applications. Researchers are encouraged to use and challenge this blueprint to advance participatory systems modeling methods.

## Appendix

Multimedia Appendix 1 Principles of participatory systems modeling, materials lists, and agendas for workshops.

## Abbreviations

BMC: Brain and Mind Centre
GP: general practitioner
