# Supplementary material for: Participatory Methods for Systems Modeling of Youth Mental Health: Implementation Protocol
Source: JMIR Res Protoc. 2022 Feb 7;11(2):e32988. doi: 10.2196/32988 (PMC8861863; doi:10.2196/32988)
Supplement: Multimedia Appendix 1 [file resprot_v11i2e32988_app1.pdf]

SUPPLEMENTARY FILE 1

Section 1: Principles of participatory systems modelling

Planning stakeholder engagement - the selection of stakeholders to include as participants, the functions they will have, their level of involvement, and at what phases of the model development process they will be involved, all require careful consideration [24, 43]. The domains of influence, skills and knowledge that each stakeholder, or group of stakeholders, will bring to the process also needs to be taken into account, together with the knowledge and skills within the modelling team, to ensure the right expertise is available to guide model development [50], prevent 'blind spots', and avoid the introduction of disciplinary bias.

Being aware of social and group dynamics – it is important to consider the motivations and intentions of stakeholders in engaging in a participatory modelling process as well as the motivations and intentions of modellers (and other professionals) in proposing the involvement of stakeholders [20]. Inclusivity in the selection of participants should be advocated for to facilitate the contribution of diverse perspectives. The social dynamics within the participant group need also to be managed, including, for example, how powerful stakeholders and special interest groups might permit, facilitate, or encourage other actors to participate, or alternatively, how they might prevent them from participating [20].

Having flexibility in the process - involving participants from the commencement of the process, defining the goals of the study and the model scope, purpose, and timeframe is important [20, 21]. Participation in the model development process itself thus becomes an important and productive part of the project. Unexpected changes in priorities should be expected and accommodated (where possible) within the process, particularly when the changes result from learning from the model [20, 21]. The priorities and focus of a commissioning agency or the project funders may have a greater influence in determining model purpose and timeline for completion, but flexibility in incorporating participant preferences and priorities within those boundaries are encouraged. Stakeholder motivation is important for the success of projects, and stakeholders may be demotivated if they are forced into a predefined protocol or procedure [21]. Striking a balance between having the project too "predetermined", i.e., too highly structured to achieve meaningful engagement, and too "unstructured", i.e., too broad and lacking focus, is important to ensure that the model can be delivered within policy relevant timeframes.

Maximising transparency and openness – being open both scientifically and socially is valuable throughout the process. Understanding the range of assumptions stakeholders hold, and designing models (and model interfaces) that facilitate the testing of alternative assumptions and their implications encourages stakeholders to integrate the models into decision making processes [21]. Throughout model development, collaborative decision making should be emphasised, allowing participants to contribute, for example, to prioritising interventions to be integrated into the model and debating which data sources are most appropriate to parameterise the model. Models need to be validated against real world data across a range of outcomes, well documented (with key assumptions, research, and data sources used made explicit), and maintained in such a way that would make them available for future use and modification if required [21].

Iterating and refining - participatory systems modelling needs to be iterative, collaborative and agile [10, 20, 21]. The active engagement of participants through the iterative development process facilitates a sense of ownership of the model where participants may come to see it as "their" model rather than a "black box" model that may not be well understood by them [10]. Participant input also helps to prevent modellers making naive assumptions that may reflect a lack of domain expertise [10] or knowledge of the social and policy environment of a given region, and lead to representation of an idealised version of a system rather than a contextually grounded one.

Encouraging learning and managing uncertainty – Models are always evolving as new evidence becomes available and new policy applications arise, and uncertainty is an important ongoing consideration [20, 21]. Models can help quantify and manage uncertainty by better understanding points of high leverage or high sensitivity in the system being modelled. Lack of completeness of data

should not be used to justify a decision not to model as methods, such as the active engagement of stakeholders to guide the parameterisation and check the logical consistency of models, explicit analysis of uncertainty and sensitivity, can be used even where there are data gaps [10]. Models have significant potential to assist learning and good decision making through the participatory process by bringing together best evidence, data and knowledge and consolidating and testing a shared hypothesis [25, 32].

Section 2: Core materials list for workshop activities

Workshop 1- conceptual mapping exercise

This activity requires:

- pre-printed and laminated elements of the model structure;
- large sheets of paper e.g., butchers' paper;
- post-it notes;
- paper tape,
- marker pens;
- audio recorders; and
- large table with space for participants to move around to view the system from different perspectives

Workshop 2 – intervention mapping exercise

This activity requires:

- pre-printed model structure on large A0 sized paper (for entire model) or A2 sized paper (for the separate model components) – the former is preferred but the latter is an appropriate alternative if A0 sized paper is not available;
- intervention questions printed on large paper for small group work;
- marker pens;
- audio recorders (1 per group); and
- large tables with space for participants to move around to view the printed systems model architecture from different perspectives.

Workshop 3 – user interaction with the model

This activity requires:

- computers with modelling software installed and model running;
- tables arranged to facilitate small group interaction; and
- sufficient printed screenshots of the user interface pages to allow participants to provide feedback on nomenclature, ranges on dials and sliders, and highlight where additional information should be provided to aid the interpretation of findings and improve general interface usability.

Section 3: Workshop agendas

Systems modelling- Workshop 1 Agenda

Right care, first time, where you live

DATE:
TIME:
VENUE:

Time	Item	Facilitator/s
08:30-09:00	Arrival, registration and tea/coffee	
09:00-09:45	**Welcome and overview of the Program aims and Objectives**<br>• Acknowledgement of Country / Acknowledgement of lived experience<br>• Welcome by host organisation<br>• Icebreaker activity<br>• Overview and aim of the project	Lead domain expert<br>Local elder/ Local lived experience representative<br>Host organisation representative<br>Systems Modelling Manager
09:45-10:15	**Session 1: Introduction to dynamic simulation modelling**<br>• What is dynamic simulation modelling and how can it help?<br>• Questions	Systems Modelling Manager
10:15-10:45	**Session 2: Introduction to the architecture, tools and symbols of systems dynamics**<br>• Introducing the tools & symbols<br>• Unfurling the simple model infrastructure<br>• Questions	Systems Modeller
10:45-11:15	**Session 3: Defining the system and outcomes of interest**<br>• Defining the problem and outcomes<br>• Discussion	Lead Domain Expert<br>Systems Modelling Manager
11:15-11:30	Morning Tea	
11:30-12:30	**Session 4: Participatory mapping exercise**<br>• Interactive pathway mapping	Systems Modelling Manager
12:30-13:30	Lunch	
13:30-14:30	**Session 4 cont: Participatory mapping exercise**	Systems Modelling Manager
14:30-15:15	**Session 5: Prioritising the interventions of interest**<br>• Interventions of interest activity	Systems Modelling Manager
15:15-15:45	**Session 6: Economics, next steps and data contribution**<br>• Introduction to the role of economic analysis<br>• Next steps and identifying data sources	Economics Lead<br>Systems Modelling Manager<br>Lead Domain Expert
15:45-16:00	**Session 7: Concluding session**<br>• Closing remarks by host organisation	Host organisation representative

Systems modelling- Workshop 2 Agenda

Right care, first time, where you live

DATE:
TIME:
VENUE:

Time	Item	Facilitator/s
08:30-09:00	Arrival, registration and tea/coffee	
09:00-09:30	**Welcome back and recap from workshop 1**<br>• Acknowledgement of Country/ Acknowledgement of lived experience<br>• Recap of the last workshop and progress since previous workshop	Local elder/ Local lived experience representative<br><br>Systems Modelling Manager
09:30-10:30	**Session 1: Presentation of the system dynamics model**<br>• Model structure to date	Systems Modeller
10:30-10:45	Morning tea	
10:45-11:15	**Session 2: Intervention mapping**<br>• Review of interventions and prioritsation	Systems Modelling Manager and Local Domain expert
11:15-12:15	**Session 3: Intervention mapping exercise**<br>• Mapping intervention mechanisms against core model structure	Systems Modelling Manager
12:15-13:15	Lunch	
13:15-14:15	**Session 3 cont: Intervention mapping exercise**	Systems Modelling Manager
14:15-15:00	**Session 4: Economic component**<br>• Overview of aims and intended approach	Economics lead
15:00-15:15	**Session 5: Concluding session**	Site representative

THE UNIVERSITY OF SYDNEY

Brain and Mind Centre

Systems modelling- Workshop 3 Agenda

Right care, first time, where you live

DATE:
TIME:
VENUE:

Time	Item	Facilitator/s
08:30-09:00	Arrival, registration and tea/coffee	
09:00-09:20	**Welcome back and recap from workshop 2 and progress update**<br>• Acknowledgement of Country/ Acknowledgement of lived experience<br>• Recap of the last workshop and progress since previous workshop	Local elder/ Local lived experience representative<br><br>Systems Modelling Manager
09:20-10:20	**Session 1: Demonstration of the systems dynamic model**<br>• Present the model structure and logic<br>• Model demonstration<br>• To explain where participant feedback has been used to refine the model.	Systems Modeller
10:20-10:35	Morning Tea	
10:35-11:35	**Session 2: User interaction with the model**<br>• Interact with model interface in groups<br>• Questions, feedback and recommendations	
11:35-12:05	**Session 3: Health economics**<br>• Presentation on status of economic analysis<br>• Discussion / feedback	Economics Lead
12:05-12:20	**Session 4: Concluding session: Next steps, closing remarks and feedback**	Systems Modeller
	Lunch (optional)	
